# Populations at Risk for Severe or Complicated Avian Influenza H5N1: A Systematic Review and Meta-Analysis

**DOI:** 10.1371/journal.pone.0089697

**Published:** 2014-03-06

**Authors:** Dominik Mertz, Tae Hyong Kim, Jennie Johnstone, Po-Po Lam, Michelle Science, Stefan P. Kuster, Shaza A. Fadel, Dat Tran, Eduardo Fernandez, Neera Bhatnagar, Mark Loeb

**Affiliations:** 1 Department of Medicine, McMaster University, Hamilton, Ontario, Canada; 2 Department of Clinical Epidemiology and Biostatistics, McMaster University, Hamilton, Ontario, Canada; 3 Michael G. DeGroote Institute for Infectious Diseases Research, McMaster University, Hamilton, Ontario, Canada; 4 Division of Infectious Diseases, Departments of Internal Medicine, Soon Chun Hyang University Seoul Hospital, Seoul, Republic of Korea; 5 Department of Microbiology, Mount Sinai Hospital, Toronto, Ontario, Canada; 6 Division of Epidemiology, Dalla Lana School of Public Health, University of Toronto, Toronto, Ontario, Canada; 7 Division of Infectious Diseases, Department of Pediatrics, The Hospital for Sick Children, University of Toronto, Ontario, Canada; 8 Division of Infectious Diseases and Hospital Epidemiology, University Hospital Zurich, University of Zurich, Zurich, Switzerland; 9 Health Sciences Library, McMaster University, Hamilton, Ontario, Canada; 10 Department of Pathology and Molecular Medicine, McMaster University, Hamilton, Ontario, Canada; Health Protection Agency, United Kingdom

## Abstract

**Background:**

Little is known about risk factors for severe outcomes in patients infected with H5N1 and no systematic review has been conducted. Understanding risk factors is an important step for prioritizing prophylaxis or treatment in the event of a pandemic.

**Objectives:**

To systematically evaluate risk factors for severe outcomes in patients with avian influenza H5N1 infection.

**Data sources:**

MEDLINE, EMBASE, CINAHL, GlobalHealth, and CENTRAL through March 2011

**Eligibility criteria for selecting studies:**

Observational studies of any design published in English, French, Spanish, German or Korean that reported on risk factor-outcome combinations of interest in participants with confirmed H5N1 infections. Outcomes considered included death, ventilator support, hospital and ICU admission, pneumonia, and composite outcomes.

**Study appraisal:**

Risk of bias was assessed using the Newcastle-Ottawa scale (NOS).

**Results:**

We identified 20 studies reporting on 999 patients infected with H5N1. The majority of studies (n = 14, 70%) were at intermediate risk of bias, i.e. 4–6 points on the NOS. Females were at increased risk of death (OR 1.75, 95% CI 1.27–2.44), while young age, in particular <5 years of age (OR 0.44, 95% CI 0.25–0.79 for death), was protective. Data on traditional risk factors was scarce and requires further studies. Another major limitation in the published literature was lack of adjustment for confounders.

**Interpretation:**

Females were at increased risk for complications following H5N1 infection while young age protected against severe outcomes. Research on traditional risk factors was limited and is required.

## Introduction

Outbreaks of highly pathogenic avian influenza H5N1 infections in poultry were first reported from Guangdong, China in 1996 [1]. Since a cluster in Hong Kong in 1997, transmission to humans has been intermittently reported and, as of April 2013, a total of 628 confirmed cases with 374 deaths of H5N1 infected patients (59.6% case fatality rate) have been reported to the World Health Organization (WHO) from 15 countries [2].

Understanding risk factors for severe outcomes is an important step in order to prioritize prophylaxis or treatment in the event of a pandemic. As part of a systematic review sponsored by the WHO, we aimed to identify risk factors for severe outcomes or complications of influenza infections. We report our findings specific to highly pathogenic avian influenza H5N1.

## Materials and Methods

The methods were reported in detail previously [3]. Briefly, we included studies reporting on at least one risk factor-outcome combination of interest irrespective of the study design. Only single-case reports were excluded, but case series reporting on patients with and without a specific outcome for a particular risk factor of interest were included. Articles based on secondary analyses of previously published data and review articles were not eligible. Outcomes of interest included community-acquired pneumonia, mortality, hospitalization, intensive care unit (ICU) admission, need for ventilator support, and composite outcomes. Studies in English, French, German, Spanish, and Korean were included.

We searched MEDLINE, EMBASE, CINAHL, Global Health, and the Cochrane Central Register of Controlled Trials (CENTRAL) up to March 29 of 2011 and reference lists of identified articles and review articles. Screening and data abstraction were conducted independently and in duplicate by pairs of reviewers using piloted and standardized forms. Two reviewers assessed the risk of bias independently using the Newcastle-Ottawa scale (NOS) [4].

A random-effects model was used to calculate summary estimates using Review Manager 5.0 (Cochrane Collaboration) [5]. Risk estimates are reported as odds ratio (OR) with 95% confidence intervals (CI). If there was a potential overlap in study populations across studies in a meta-analysis, studies were excluded in a sensitivity analysis in order to minimize any effect of such overlap. The I^2^ statistics was used to evaluate heterogeneity [6]. Two a priori defined subgroup analysis based on the place of enrollment (community versus hospital versus ICU population) and risk of bias in included studies were conducted in the presence of significant heterogeneity defined by I^2^>60% [7]. Funnel plots were visually interpreted to assess publication bias but no formal statistical tests were conducted due to the small number of studies for the vast majority of risk factor-outcome combinations.

The World Health Organization (WHO) funded the study. The protocol was reviewed by the WHO and the WHO's suggestion were incorporated into the protocol.

## Results

**Figure 1:**
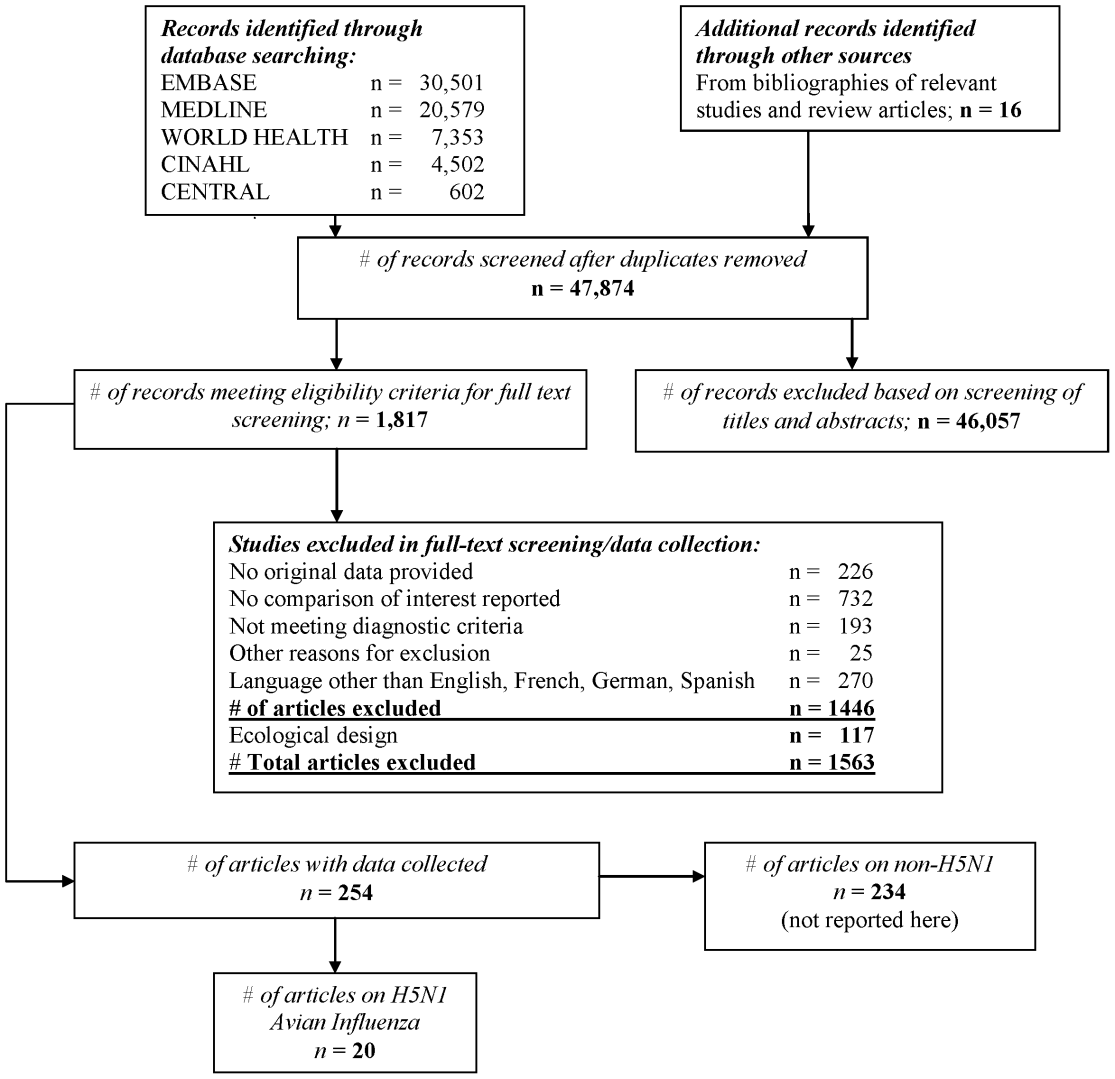
Flow diagram of included and excluded studies.

Out of 47,874 titles and abstracts screened, 20 studies [8], [9], [10], [11], [12], [13], [14], [15], [16], [17], [18], [19], [20], [21], [22], [23], [24], [25], [26], [27] that included 999 patients reported risk factors for severe outcomes for H5N1 avian influenza (Figure 1). All studies were cohort studies and published in English between 1998 and 2010. Evidence of H5N1 influenza infection was by laboratory confirmation in all studies. Only two studies reported on cases outside of Asia.

As assessed by the Newcastle-Ottawa scale, the majority of studies were at intermediate risk of bias (i.e. 4–6 points; n = 14, 70%) while 4 (20%) were at highest risk of bias (i.e. 3 or less points) and 2 (10%) at lowest risk of bias (i.e. 7–9 points). Although the adjusted odds ratios were reported in only one study, the use of adjusted odds ratios instead of the crude odds ratios would not have changed our assessment of the risk factors.

**Figure 2:**
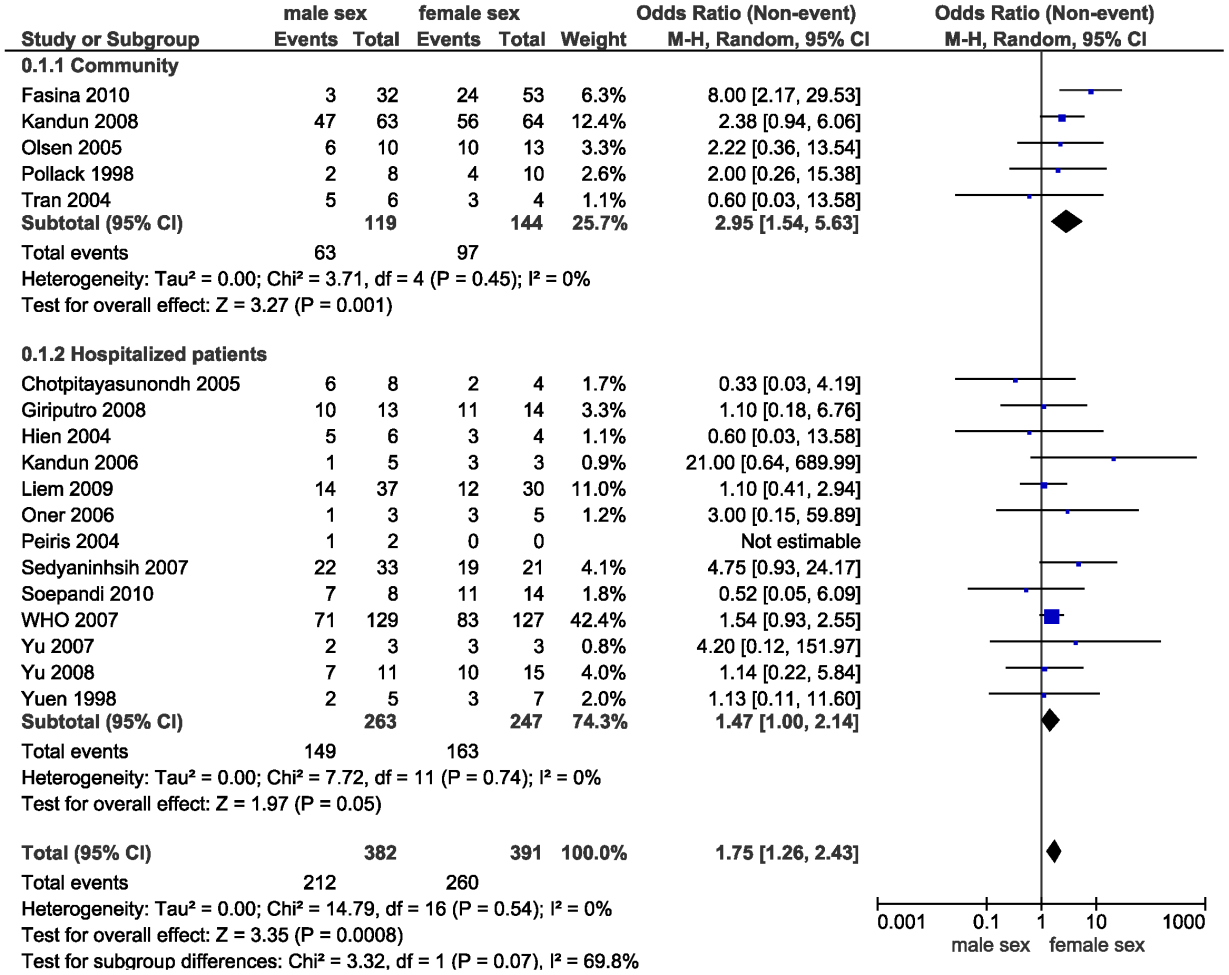
Forest plot comparing mortality in females as compared to males.

Female sex was associated with higher all-cause mortality (OR 1.75, 95% 1.27–2.44) (Figure 2, Table 1). We also found a non-significant trend towards higher rates of pneumonia (OR 3.03, 95% CI 0.91–10.00), ICU admission (OR 3.70, 95% CI 0.33–50.00), and need for ventilator support (OR 2.94, 95% CI 0.43–20.00) in women. Because overlap in study populations was likely across some studies for mortality and pneumonia outcomes, a sensitivity analysis was conducted excluding studies with a potential overlap. The findings were similar (Table 1).

**Table 1:** Summary estimates of risk-factor–outcome comparisons during H5N1 Avian influenza with odds ratio (95% confidence interval), I^2^, and number of included studies (n); where applicable, sensitivity analysis in *italic* fonts.

	*Pneumonia*	*ICU admission*	*Ventilator support*	*All-cause mortality*
Female sex	3.03 (0.91–10.00), 0%, n = 4	3.70 (0.33–50.00), n/a, n = 1	2.94 (0.43–20.00), 0%, n = 3	1.75*(1.27–2.44), 0%, n = 17
	*5.00 (0.91–33.33), 0%, n = 2*			*2.27* * *(1.12–4.55), 50%, n = 4*
Elderly vs. non elderly adults	n/a	n/a	n/a	1.04 (0.12–9.08), 0%, n = 2
Paediatric vs. adults	0.16*(0.03–0.86), 0%, n = 3	0.50 (0.02–12.90), n/a, n = 1	0.62 (0.09–4.45), 0%, n = 3	0.72 (0.34–1.51), 71%, n = 14
	*0.12 (0.01–1.03), 0%, n = 2*			*0.21 (0.03–1.41), 87%, n = 4*
Paediatric vs.	0.12 (0.01–1.03), 0%, n = 2	0.50 (0.02–12.90), n/a, n = 1	0.93 (0.10–8.27), 0%, n = 2	0.48 (0.13–1.79), 73%, n = 6
non-elderly adults				*0.25 (0.04–1.55), 87%, n = 3*
<5 y in paediatric	0.27 (0.01–7.59), 50%, n = 2	n/a	0.11 (0.00–3.35), n/a, n = 1	0.44*(0.25–0.79), 0%, n = 7
population	*0.06 (0.00–1.23),n/a, n = 1*			*0.42* * *(0.18–0.97), 0%, n = 2*
<2 y in paediatric population	0.71 (0.02–22.34), n/a, n = 1	n/a	n/a	1.13 (0.03–37.44), n/a, n = 1
Any risk factor or	1.71 (0.35–8.36), 0%, n = 2	0.60 (0.05–6.79), n/a, n = 1	n/a	1.04 (0.20–5.35), 9%, n = 2
co-morbidity	*1.43 (0.18–11.1),n/a, n = 1*			
Smoking	9.00 (0.30–271.65), n/a, n = 1	5.00 (0.15–166.59), n/a, n = 1	0.58 (0.06–5.55), 0%, n = 2	0.56 (0.05–5.55), 0%, n = 2

*: statistical significance, ICU: admission to intensive care unit, y: years; sensitivity analysis in *italic*: exclusion of studies with a potential overlap in patient population.

**Figure 3:**
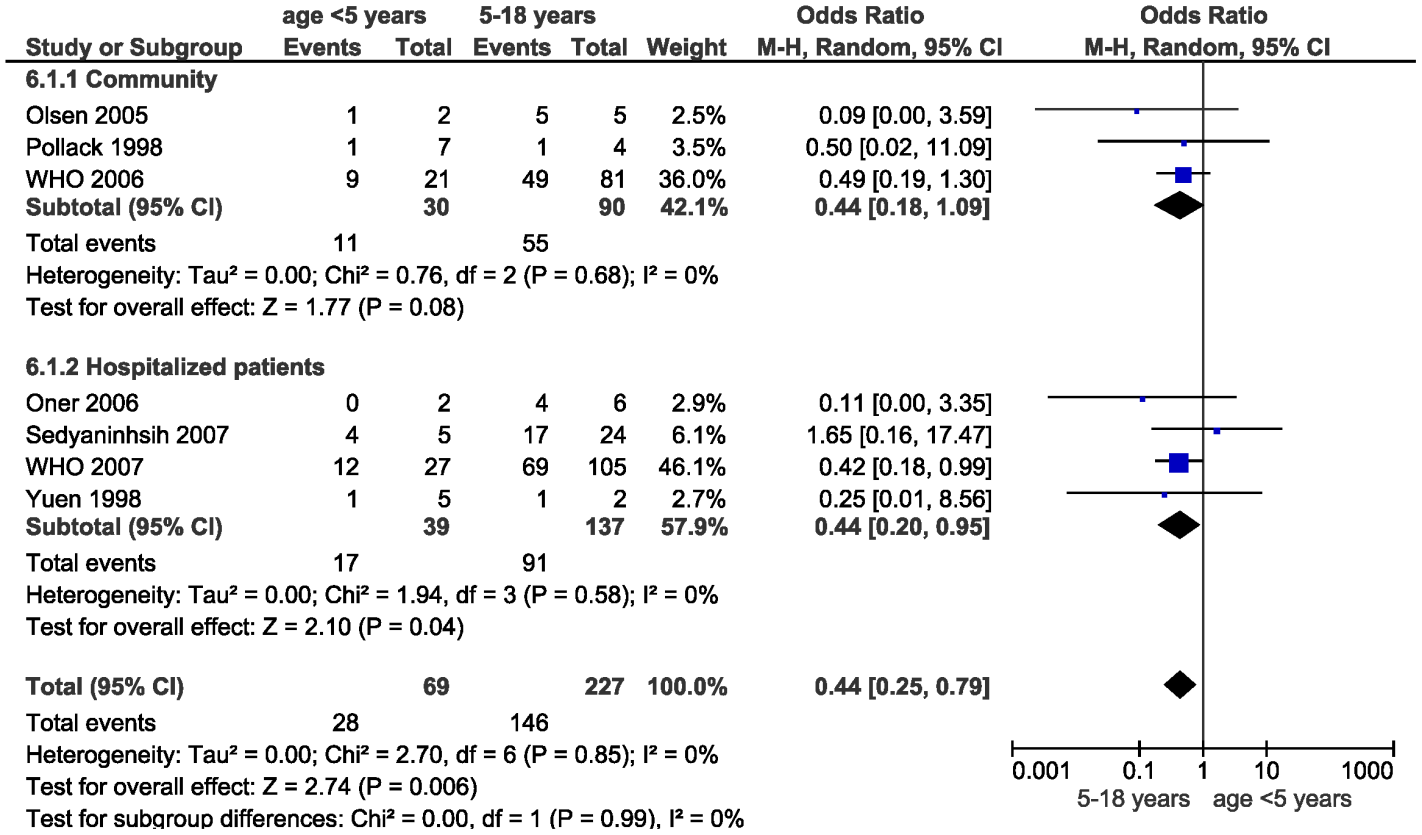
Forest plot comparing mortality in children less than 5 years of age to 5–18 years of age.

We found no evidence that older age was a risk factor for more severe outcomes when comparing >65 years of age to non-elderly adults (OR for all-cause mortality 1.04, 95% CI 0.12–9.08). Children <18 years of age were at lower risk of developing pneumonia (OR 0.16, 95% CI 0.03–0.86) when compared to adult patients. There was a non-significant tendency towards lower mortality rates (0.48, 95% CI 0.13–1.79) and lower risk of pneumonia (OR 0.12, 95% CI 0.01–1.03) when compared to non-elderly adults. We found that children of <5 years of age were at lower risk of death when compared to older children 5–18 years of age (OR 0.44, 0.25–0.79; Figure 3) with a non-significant trend towards a lower likelihood of developing pneumonia or need ventilator support.

Only two studies reported on the presence of co-morbidities as potential traditional risk factors. Although no significant differences were found, there was a non-significant tendency towards increased risk of pneumonia in the presence of co-morbidities (OR 1.71, 95% CI 0.35–8.36). No significant differences in outcomes were found for smoking.

The only comparison with significant heterogeneity was for all-cause mortality when comparing paediatric patients to non-elderly adults (I^2^ = 73%). A subgroup analysis excluding studies at highest risk of bias (Newcastle-Ottawa scale of 3 or lower) reduced the I^2^ to 0% with a smaller overall effect (OR 0.90, 95% CI 0.51–1.57 versus OR 0.48, 95% CI 0.13–1.79) in the primary analysis.

## Discussion

We found that female sex was associated with worse outcomes following H5N1 infection and that children, particularly those <5 years old, had better outcomes. Data on underlying co-morbidities as risk factors for severe outcomes were scarce and therefore there is no evidence demonstrating that co-morbidities affect the outcome in patients infected with H5N1.

At the time of our literature search, 539 human cases with H5N1 infections from 15 countries had been reported to WHO [28]. In the 20 studies identified in our systematic review, a total of 999 cases were reported. While it seems unlikely that a large number of confirmed H5N1 infections have not been reported to WHO [29], the excess in cases in combination with identical time periods and regions reported in included studies indicates overlap in patient populations reported in the literature. In particular, there was an overlap between two large WHO reports (Appendix A, references 11 and 13) and studies published by local investigators. In a sensitivity analysis we excluded studies that appeared to have included some participants reported in another included study, the results were in keeping with the primary analyses. Since the overlap in participants between studies was never 100%, the full dataset was presented as the primary analysis.

Female sex was associated with a higher mortality risk as well as with a non-significant trend for other severe outcomes. Our findings are in line with a report from Egypt where the H5N1 case fatality rate was 49% in females compared to 8% in males [30]. Of note, the overall mortality rate was lower in Egypt as compared to mortality rates reported from other countries (34 versus 66%) [30]. A higher mortality in females was also found in a review of 294 cases occurring between 2006 and 2010 published by Fiebig et al. [29]. Women infected with H5N1 were older [29] and most of the affected women were housewives taking care of poultry [31]. In contrast, the males were largely pre-school boys that got exposed while playing outdoors [31]. An exposure to a greater inoculum is one possibility for the worse outcomes in women. Another is that the sex differences were confounded by age: females were likely exposed at an older age than boys, and it was shown that younger age, in particular <5 years of age, was associated with a lower risk for severe outcomes. This is in keeping with the findings in the above mentioned study by Fiebig et al. There, the authors found an even more pronounced difference with five to six times lower odds for death for children up to 9 years of age as compared to a number of other age groups [29].

In contrast to non-H5N1 influenza infections where older age was shown to be a risk factor for severe outcomes for seasonal influenza as well as during the 2009 H1N1 pandemic [3], this does not seem to be true for H5N1 infections. One can hypothesize that previous exposure to other subtypes may have offered protection against H5N1 [32].

One limitations of this systematic review is the potential for reporting bias. In particular asymptomatic patients or patients with minor symptoms were likely underrepresented in the cases reported. In fact, a 1 to 2% infection rate in those exposed was found in serological studies which would result in much higher number of cases than reported [33], which in turn is regarded to be an overestimate by others [34]. Adjusted risk estimates also were not available. Thus, modifying risk factors such as differing time to oseltamivir treatment or the availability of health care may have biased outcomes [29], [35]. Also, we did not consider articles in Chinese or other languages spoken in the regions where H5N1 is endemic. However, we did not find any relevant articles on H5N1 when screening titles and abstracts of the 50 Chinese articles that were excluded. Among the remaining 220 articles that were excluded due to language, we only identified three review articles on H5N1 when searching for the key words “avian” or “H5N1” in the title. Thus, we believe that we did not miss any relevant articles on Avian Influenza in the databases that we searched due to restrictions on language. Finally, sample sizes for some rarely reported risk factor-outcome comparisons were small explaining the low precision and likely the lack of statistical significance for some of these trends that we noted.

In summary, we found evidence that females were at increased risk for complications following H5N1 infection while young age protected against severe outcomes. Research on traditional risk factors is scarce and is required.

## Supporting Information

Checklist S1 PRISMA checklist.
(DOC)
